# Supplementary figures and images for: Effects of walking impairment on mental health burden, health risk behavior and quality of life in patients with intermittent claudication: A cross-sectional path analysis
Source: PLoS One. 2022 Sep 1;17(9):e0273747. doi: 10.1371/journal.pone.0273747 (PMC9436130; doi:10.1371/journal.pone.0273747)

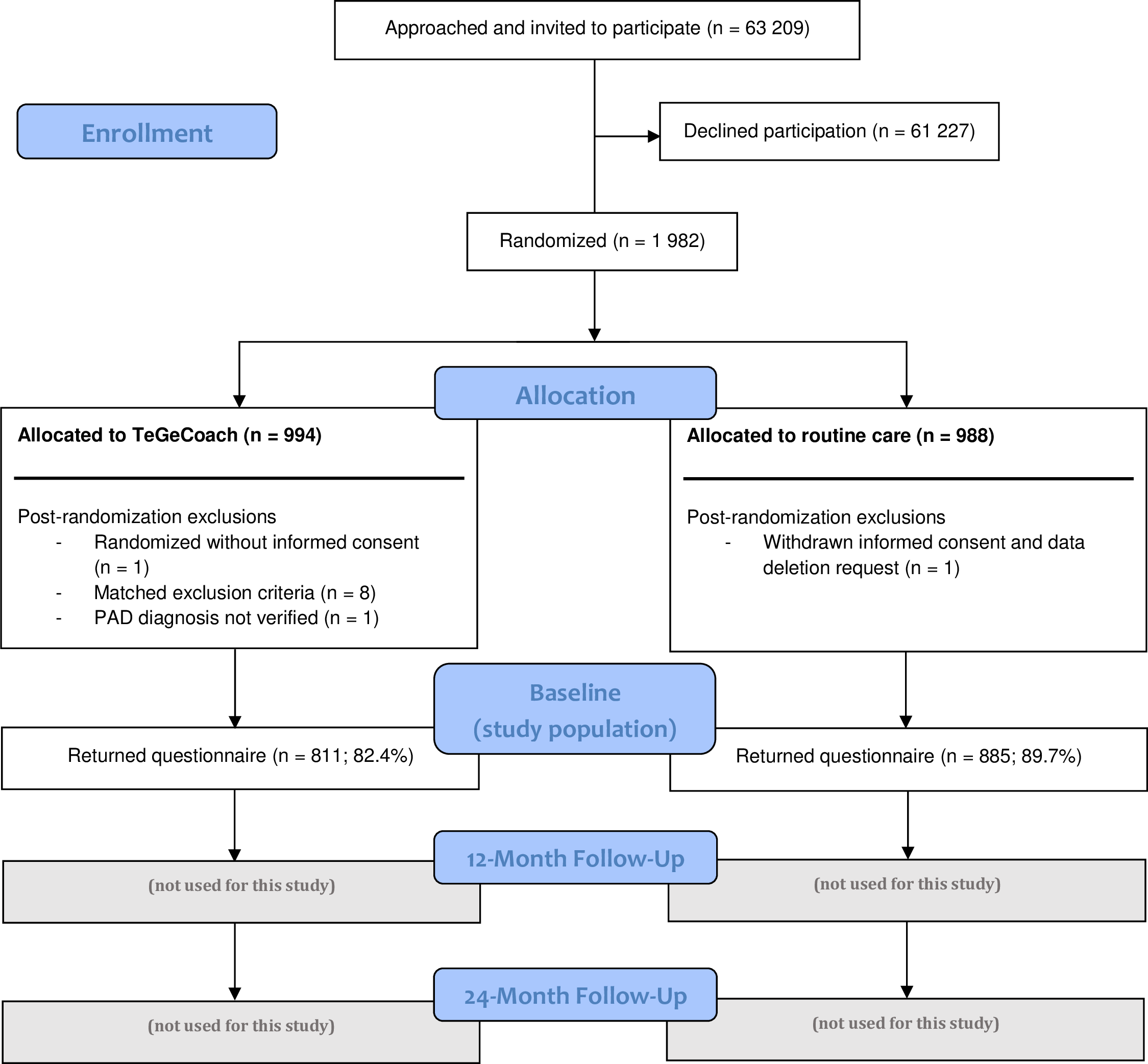

Supplement: S1 Fig — (TIF) [file pone.0273747.s001.tif]
