# Supplementary material for: Effects of walking impairment on mental health burden, health risk behavior and quality of life in patients with intermittent claudication: A cross-sectional path analysis
Source: PLoS One. 2022 Sep 1;17(9):e0273747. doi: 10.1371/journal.pone.0273747 (PMC9436130; doi:10.1371/journal.pone.0273747)
Supplement: S2 File — (PDF) [file pone.0273747.s003.pdf]

Model iterations

Proposed model (i.e. before respecification, Model 1)

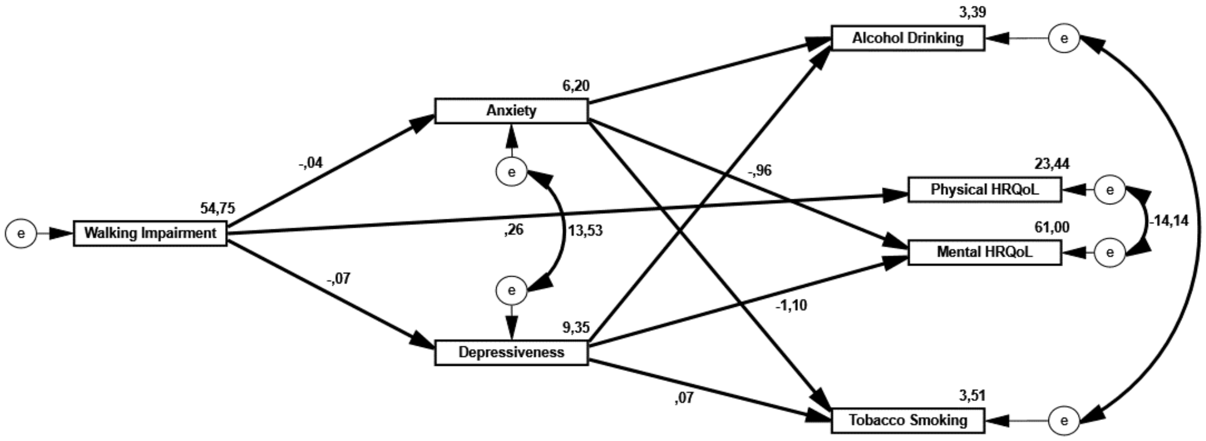

Goodness-of-fit parameters: CMIN/DF = 10.223; TLI = .942; CFI = .981; RMSEA = .074

Regression Weights

			Estimate	S.E.	C.R.	P
Anxiety	<---	Walking Impairment	-,040	,004	-9,920	***
Depressive symptoms	<---	Walking Impairment	-,069	,004	-15,597	***
physical HRQoL	<---	Walking Impairment	,264	,007	36,121	***
mental HRQoL	<---	Depressive symptoms	-1,103	,062	-17,689	***
mental HRQoL	<---	Anxiety	-,960	,071	-13,496	***
Alcohol Drinking	<---	Anxiety	-,021	,024	-,871	,384
Tobacco Smoking	<---	Depressive symptoms	,070	,030	2,368	,018
Tobacco Smoking	<---	Anxiety	,063	,034	1,860	,063
Alcohol Drinking	<---	Depressive symptoms	-,029	,021	-1,411	,158

Covariances

			Estimate	S.E.	C.R.	P
e(Depressive symptoms)	<-->	e(Anxiety)	13,532	,535	25,294	***
e(mental HRQoL)	<-->	e(physical HRQoL)	-14,136	1,394	-10,138	***
e(Alcohol Drinking)	<-->	e(Tobacco Smoking)	,192	,202	,948	,343

Model 2

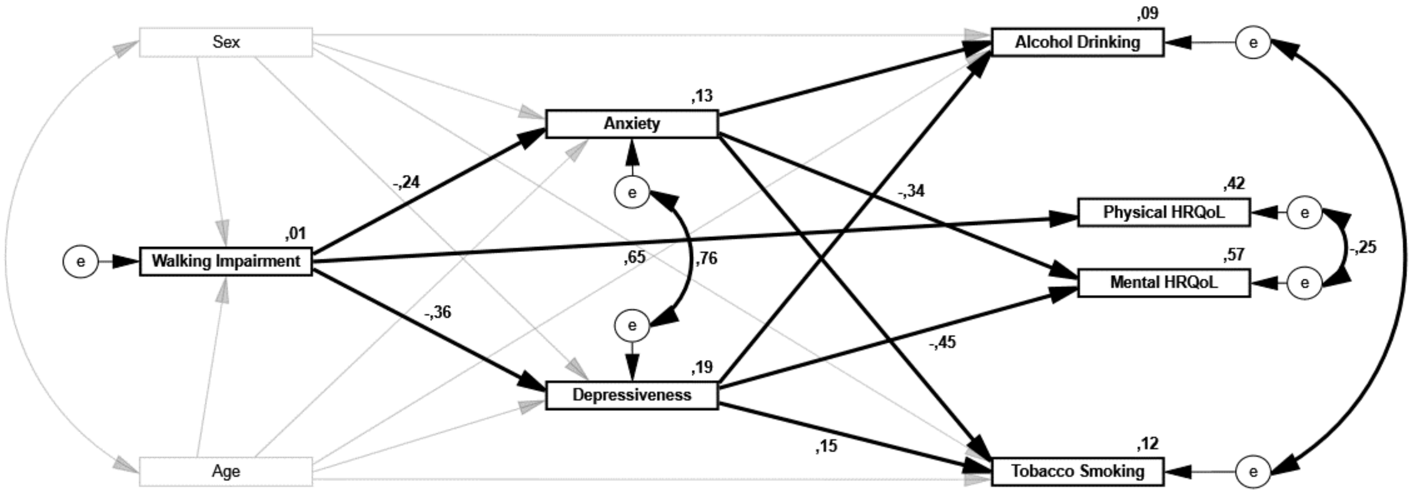

Goodness-of-fit parameters: CMIN/DF = 7.506; TLI = .939; CFI = .982; RMSEA = .062

Regression Weights

			Estimate	S.E.	C.R.	P
Walking Impairment	<---	Sex	5,370	1,261	4,257	***
Walking Impairment	<---	Age	-,156	,068	-2,294	,022
Anxiety	<---	Walking Impairment	-,040	,004	-10,395	***
Depressive symptoms	<---	Sex	-,960	,224	-4,282	***
Depressive symptoms	<---	Age	-,120	,012	-9,963	***
Depressive symptoms	<---	Walking Impairment	-,069	,004	-16,104	***
Anxiety	<---	Sex	-,792	,203	-3,910	***
Anxiety	<---	Age	-,120	,011	-11,045	***
physical HRQoL	<---	Walking Impairment	,264	,007	36,121	***
mental HRQoL	<---	Depressive symptoms	-1,103	,062	-17,689	***
mental HRQoL	<---	Anxiety	-,960	,071	-13,496	***
Alcohol Drinking	<---	Sex	1,506	,123	12,239	***
Tobacco Smoking	<---	Age	-,061	,010	-6,120	***
Alcohol Drinking	<---	Anxiety	-,022	,023	-,983	,325
Tobacco Smoking	<---	Depressive symptoms	,073	,029	2,505	,012
Alcohol Drinking	<---	Age	-,022	,007	-3,263	,001
Tobacco Smoking	<---	Anxiety	,042	,033	1,252	,210
Alcohol Drinking	<---	Depressive symptoms	-,014	,020	-,684	,494
Tobacco Smoking	<---	Sex	,395	,180	2,191	,028

Covariances

			Estimate	S.E.	C.R.	P
Sex	<-->	Age	,424	,099	4,291	***
e(Depressive symptoms)	<-->	e(Anxiety)	12,208	,490	24,906	***
e(mental HRQoL)	<-->	e(physical HRQoL)	-14,136	1,394	-10,138	***
e(Alcohol Drinking)	<-->	e(Tobacco Smoking)	,032	,189	,171	,864

Model 3

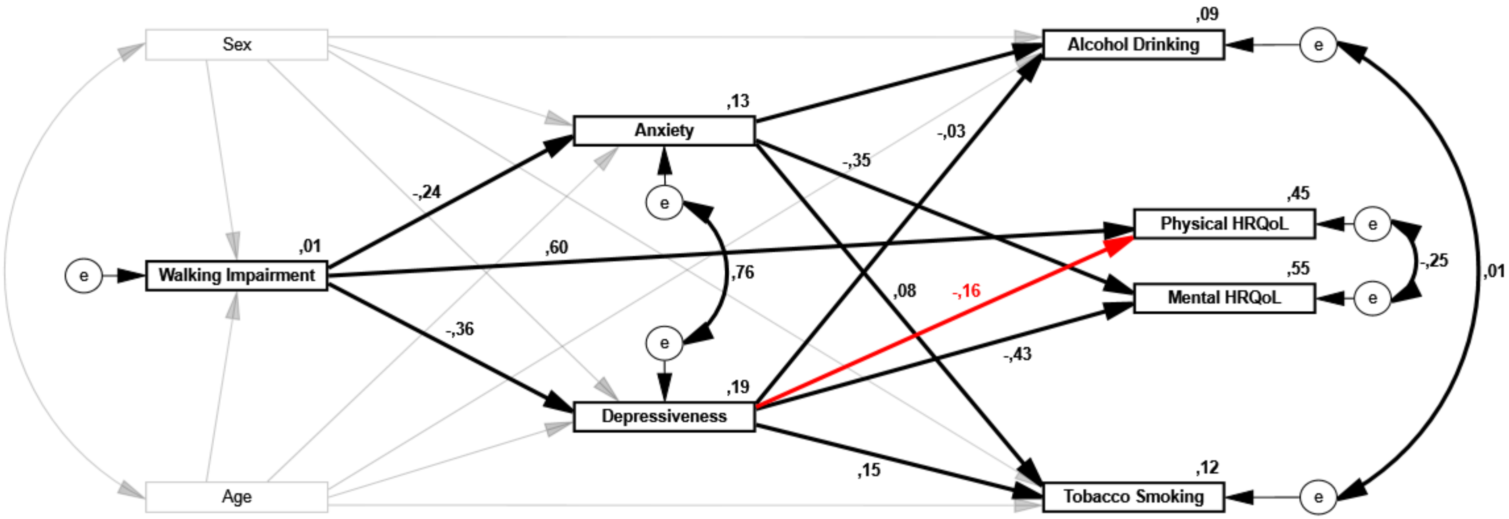

Goodness-of-fit parameters: CMIN/DF = 2.743; TLI = .984; CFI = .996; RMSEA = .032

Regression Weights

			Estimate	S.E.	C.R.	P
Walking Impairment	<---	Sex	5,370	1,261	4,257	***
Walking Impairment	<---	Age	-,156	,068	-2,294	,022
Anxiety	<---	Walking Impairment	-,040	,004	-10,395	***
Depressive symptoms	<---	Sex	-,960	,224	-4,282	***
Depressive symptoms	<---	Age	-,120	,012	-9,963	***
Depressive symptoms	<---	Walking Impairment	-,069	,004	-16,104	***
Anxiety	<---	Sex	-,792	,203	-3,910	***
Anxiety	<---	Age	-,120	,011	-11,045	***
physical HRQoL	<---	Walking Impairment	,243	,008	31,732	***
mental HRQoL	<---	Depressive symptoms	-1,031	,063	-16,388	***
mental HRQoL	<---	Anxiety	-,955	,071	-13,434	***
Alcohol Drinking	<---	Sex	1,506	,123	12,239	***
Tobacco Smoking	<---	Age	-,061	,010	-6,120	***
Alcohol Drinking	<---	Anxiety	-,022	,023	-,983	,325
Tobacco Smoking	<---	Depressive symptoms	,073	,029	2,505	,012
Alcohol Drinking	<---	Age	-,022	,007	-3,263	,001
Tobacco Smoking	<---	Anxiety	,042	,033	1,252	,210
Alcohol Drinking	<---	Depressive symptoms	-,014	,020	-,684	,494
Tobacco Smoking	<---	Sex	,395	,180	2,191	,028
physical HRQoL	<---	Depressive symptoms	-,328	,040	-8,116	***

Covariances: (Group number 1 - Default model)

			Estimate	S.E.	C.R.	P
Sex	<-->	Age	,424	,099	4,291	***
e(Depressive symptoms)	<-->	e(Anxiety)	12,208	,490	24,906	***
e(mental HRQoL)	<-->	e(physical HRQoL)	-13,726	1,366	-10,047	***
e(Alcohol Drinking)	<-->	e(Tobacco Smoking)	,032	,189	,171	,864

Model 4 (final model)

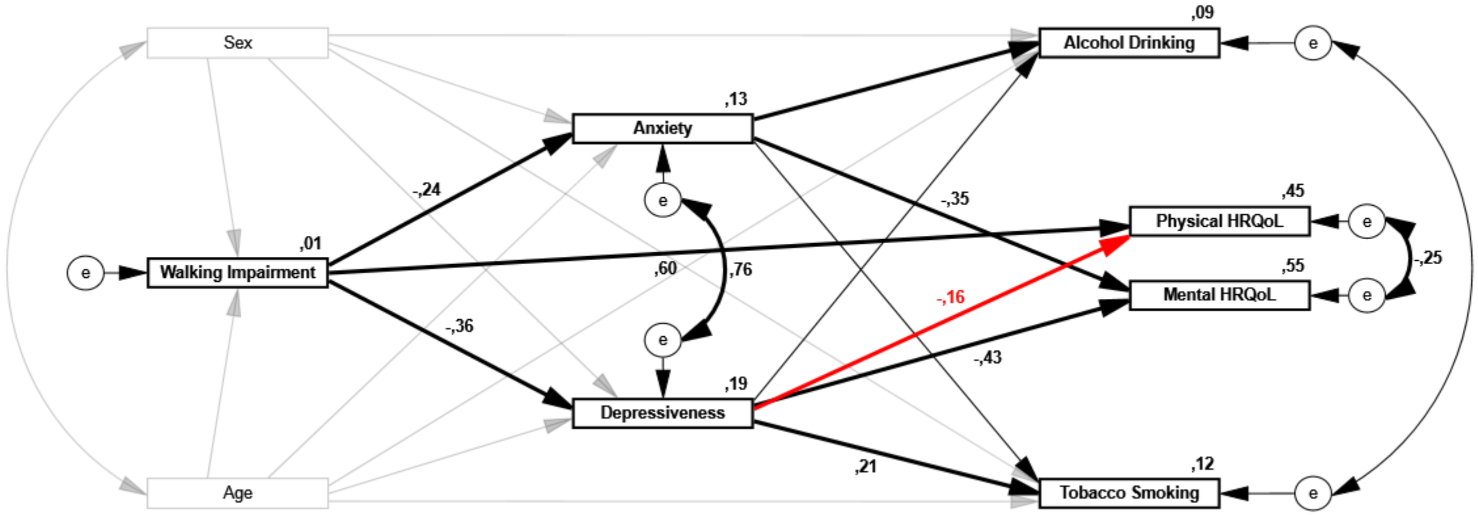

Goodness-of-fit parameters: CMIN/DF = 2.345; TLI = .987; CFI = .996; RMSEA = .028

Regression Weights

			Estimate	S.E.	C.R.	P
Walking Impairment	<---	Sex	5,369	1,261	4,257	***
Walking Impairment	<---	Age	-,156	,068	-2,293	,022
Anxiety	<---	Walking Impairment	-,040	,004	-10,395	***
Depressive symptoms	<---	Sex	-,960	,224	-4,282	***
Depressive symptoms	<---	Age	-,120	,012	-9,964	***
Depressive symptoms	<---	Walking Impairment	-,069	,004	-16,104	***
Anxiety	<---	Sex	-,792	,203	-3,909	***
Anxiety	<---	Age	-,120	,011	-11,048	***
physical HRQoL	<---	Walking Impairment	,243	,008	31,732	***
mental HRQoL	<---	Depressive symptoms	-1,031	,063	-16,388	***
mental HRQoL	<---	Anxiety	-,955	,071	-13,434	***
Alcohol Drinking	<---	Sex	1,512	,123	12,315	***
Tobacco Smoking	<---	Age	-,063	,010	-6,372	***
Alcohol Drinking	<---	Anxiety	-,035	,014	-2,420	,016
Tobacco Smoking	<---	Depressive symptoms	,101	,018	5,549	***
Alcohol Drinking	<---	Age	-,022	,007	-3,250	,001
Tobacco Smoking	<---	Anxiety	,000			
Alcohol Drinking	<---	Depressive symptoms	,000			
Tobacco Smoking	<---	Sex	,381	,180	2,114	,035
physical HRQoL	<---	Depressive symptoms	-,328	,040	-8,116	***

Covariances

			Estimate	S.E.	C.R.	P
Sex	<-->	Age	,424	,099	4,290	***
e(Depressive symptoms)	<-->	e(Anxiety)	12,208	,490	24,906	***
e(mental HRQoL)	<-->	e(physical HRQoL)	-13,726	1,366	-10,047	***
e(Alcohol Drinking)	<-->	e(Tobacco Smoking)	,000			
